# Protective Antigen-Specific Memory B Cells Persist Years after Anthrax Vaccination and Correlate with Humoral Immunity

**DOI:** 10.3390/toxins6082424

**Published:** 2014-08-13

**Authors:** Lori Garman, Kenneth Smith, A. Darise Farris, Michael R. Nelson, Renata J. M. Engler, Judith A. James

**Affiliations:** 1 Department of Arthritis and Clinical Immunology, Oklahoma Medical Research Foundation, 825 NE 13th Street, Oklahoma City, OK 73104, USA; E-Mails: Lori-Garman@omrf.org (L.G.); Ken-Smith@omrf.org (K.S.); Darise-Farris@omrf.org (A.D.F.); 2 Department of Microbiology and Immunology, University of Oklahoma Health Sciences Center, 940 Stanton L. Young Blvd, Oklahoma City, OK 73104, USA; 3 Military Vaccine Agency-Vaccine Healthcare Centers Network, Walter Reed National Military Medical Center, 8901 Rockville Pike, Bethesda, MD 20889, USA; E-Mails: michael.r.nelson.mil@health.mil (M.R.N.); renata.j.engler.mil@health.mil (R.J.M.E.); 4 Department of Medicine, University of Oklahoma Health Sciences Center, 1000 Stanton L. Young Blvd, Oklahoma City, OK 73104, USA

**Keywords:** Anthrax Vaccine Adsorbed, cellular immunity, lethal toxin neutralization, protective antigen

## Abstract

Anthrax Vaccine Adsorbed (AVA) generates short-lived protective antigen (PA) specific IgG that correlates with *in vitro* toxin neutralization and protection from *Bacillus anthracis* challenge. Animal studies suggest that when PA-specific IgG has waned, survival after spore challenge correlates with an activation of PA-specific memory B cells. Here, we characterize the quantity and the longevity of AVA-induced memory B cell responses in humans. Peripheral blood mononuclear cells (PBMCs) from individuals vaccinated ≥3 times with AVA (*n* = 50) were collected early (3–6 months, *n* = 27) or late after their last vaccination (2–5 years, *n* = 23), pan-stimulated, and assayed by ELISPOT for total and PA-specific memory B cells differentiated into antibody secreting cells (ASCs). PA-specific ASC percentages ranged from 0.02% to 6.25% (median: 1.57%) and did not differ between early and late post-vaccination individuals. PA-specific ASC percentages correlated with plasma PA-specific IgG (*r* = 0.42, *p* = 0.03) and toxin neutralization (*r* = 0.52, *p* = 0.003) early post vaccination. PA-specific ASC percentages correlated with supernatant anti-PA both early (*r* = 0.60, *p* = 0.001) and late post vaccination (*r* = 0.71, *p* < 0.0001). These data suggest PA-specific memory B cell responses are long-lived and can be estimated after recent vaccination by the magnitude and neutralization capacity of the humoral response.

## 1. Introduction

The generation of immunological memory to T cell-dependent antigens results in both humoral and cellular immunity. In this process, naïve B cells enter the germinal center reaction and exit as antibody secreting cells (ASCs; plasmablasts or long-lived plasma cells) or memory B cells [1]. Together, ASCs and memory B cells are responsible for maintaining humoral immunity generated by vaccination [2]. ASCs are terminally differentiated and maintain humoral immunity by actively producing antibody. In contrast, memory B cells are a quiescent population that can, on activation, differentiate into ASCs up to decades after original stimulation [1,3,4]. Whether ASCs and memory B cells represent independently controlled memory populations in humans is unclear. Humoral immunity and memory B cell levels are correlated in smallpox vaccinees [5], but not in tetanus immunization, wasp venom immunotherapy, or prior malaria infection [6,7].

Like most vaccines [8,9], the correlate of protection for Anthrax Vaccine Adsorbed (AVA) is antibodies directed toward its primary immunogen, protective antigen (PA) [10]. PA-specific antibody level also correlates strongly with anthrax specific *in vitro* lethal toxin (LT) neutralization activity (LTNA). In turn, LTNA has been demonstrated to be predictive of survival in several animal models, including a non-human primate *Bacillus anthracis* spore challenge model [11]. However, a subset of vaccinated military personnel may not be adequately protected in the event of spore exposure [12,13]. Among individuals vaccinated three or more times with AVA and receiving their most recent vaccination within the year prior to sample collection (*n* = 1422), 17.6% do not have significant plasma anti-PA IgG (<10 µg/mL), and 30.9% neutralize toxin no better than unvaccinated controls (<12% viability) [12,13].

In mouse and *Rhesus macaque* studies in which antibody titers to PA are allowed to decline prior to challenge with anthrax spores, subsets of animals survive challenge and demonstrate evidence of memory B cell activation in the form of increased post-challenge PA antibody levels [11,14]. To determine if vaccinated individuals with low levels of LT-neutralizing anti-PA IgG possess PA immunity through memory B cells, we first measured the persistence of PA-specific memory B cells following AVA vaccination in a real-world cohort. In addition, we assessed the ability of anti-PA IgG and LTNA to function as a surrogate for memory B cell immunity. We hypothesized that antibody levels are maintained by long-lived plasma cells independent of memory B cells; while antibody and memory B cells may correlate early post-vaccination due to a relatively good or poor germinal center reaction, we expected the correlation of memory B cells and antibody levels to decline late post vaccination.

## 2. Results and Discussion

### 2.1. Functional PA-Specific Memory B Cells Are Retained for Years Following Vaccination

**Figure 1:**
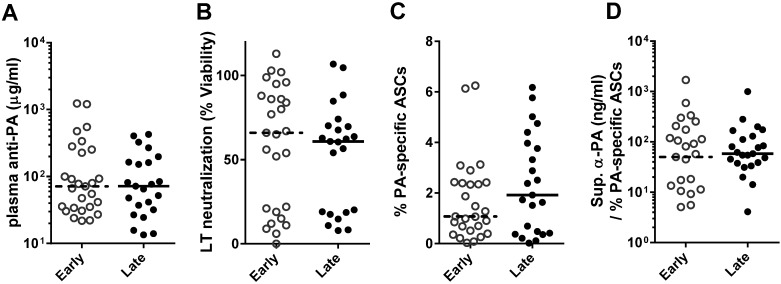
Quantity and quality of memory B cells are similar early and late post-vaccination in individuals with similar humoral responses. Humoral responses of AVA-vaccinated individuals collected early (3–6 months) or late (2–5 years) after their last vaccination did not differ by protective antigen (PA) ELISA (**A**, *p* = 0.21) and *in vitro* lethal toxin (LT) neutralization (**B**, *p* = 0.43). Similarly, PA-specific ASCs (**C**) and anti-PA IgG per ASC in culture supernatants (**D**) were not significantly different (*p* = 0.52, *p* = 0.41, respectively) between early and late post-vaccination individuals. Groups were compared by two-tailed Mann-Whitney *U*. Each symbol indicates a single individual, bars indicate median values.

To assess the longevity of memory B cell immunity, we chose individuals (*n* = 50) from our previously published AVA-vaccinated cohort [12,13] that had been vaccinated at least three times (range: 3–9; mean: 5.1) and differed in humoral response and time since their most recent vaccination. Specifically, individuals were chosen with low (<100 µg/mL) plasma anti-PA IgG and low LTNA (<25%), high (>150 µg/mL) anti-PA IgG and high LTNA (>50%), and low anti-PA IgG and high LTNA (Table 1). In addition, these individuals were sampled either early post-vaccination (3–6 months, *n* = 27) or late post-vaccination (2–5 years, *n* = 23). While anti-PA IgG typically declines rapidly in AVA-vaccinated individuals following vaccination [12,13], individuals were selected such that median plasma anti-PA IgG (Figure 1A) and LTNA (Figure 1B) were not significantly different between early and late post-vaccination individuals. While individuals in this cohort ranged in number of vaccinations (3–9; Table 1), number of vaccinations was not significantly correlated with anti-PA IgG, LTNA, or PA-specific ASCs (data not shown) in this group of individuals.

**Table 1:** Demographic and vaccination history information of Anthrax Vaccine Adsorbed (AVA)-vaccinated individuals.

	Early post-vaccination individuals (*n* = 27)	Late post-vaccination individuals (*n* = 23)
**Age at collection**		
Average (SD)	26.2 (5.2)	34.4 (5.6)
Median (range)	25 (19–41)	35 (23–45)
**Years since last vaccination**		
Average (SD)	0.39 (0.09)	3.09 (0.68)
Median (range)	0.42 (0.25–0.5)	3.05 (2.10–4.33)
**Number of vaccinations**		
Average (SD)	4.4 (1.2)	6 (1.4)
Median (range)	4 (3–7)	6 (4–9)

PBMCs from the same blood sample as the characterized plasma were pan-stimulated for 6 days to allow memory B cells to differentiate into antibody-secreting cells (ASCs), and the frequency of PA-specific ASCs as a percentage of the total IgG-secreting ASCs were assessed. Similar to smaller, previously published human studies [3,15], PA-specific ASC percentages were highly variable (0.02%–6.25%) with a median of 1.57%.

Our previous work has demonstrated that anti-PA IgG declines rapidly [12,13]. However, memory B cells have been known to persist for decades after vaccination even in the absence of antibody levels [3]. Consistent with these results, we expected the apparent rate of decline of anti-PA IgG to be steeper than the decline of PA-specific memory B cells following AVA. We therefore hypothesized that the late post vaccination individuals would have had extremely high levels of anti-PA if sampled early post vaccination, and would have higher levels of PA-specific ASCs than early post vaccination individuals. Contrary to our hypothesis, PA-specific ASCs did not differ between early and late post vaccination individuals (medians: 1.07%, 1.92%; Figure 1C).

The quality of memory B cells, or how much anti-PA IgG each memory B cell made, was approximated for each individual by dividing the amount of PA-specific IgG present in day 6 culture supernatants by the frequency of PA-specific ASCs. The quality of memory B cells was similar in early and late post-vaccination individuals (medians: 50.1 ng/mL/%, 58.4 ng/mL/%; Figure 1D). Unvaccinated controls had medians of 0.07% PA-specific ASCs and 2.1 ng/mL supernatant anti-PA IgG (data not shown). Together, these data indicate that PA-specific ASCs can persist at high levels for years following vaccination. The nanogram quantities of anti-PA IgG produced by these few memory B cells, differentiated into ASCs, suggest PA-specific memory B cells can function as a reservoir of antibody in the event of exposure.

### 2.2. Functional PA-Specific Memory B Cells Are Retained for Years Following Vaccination

Anthrax infection has an incubation period of 1–14 days [16], and inhalational anthrax cases usually lead to death or resolution within 5 days after exposure, or 72 h after symptoms present [17]. In human anthrax vaccination, PA-specific lymphocytes can persist for as long as 15 years after vaccination, long after anti-PA titers have diminished, and could provide protection in individuals that have not received yearly boosters [18]. The time course of infection makes it feasible that anthrax exposure could activate memory B cells and quickly regenerate protective antibody titers; *i.e.*, PA-specific memory B cells could potentially function as a distinct reservoir of anthrax immunity that could be activated in the event of exposure. However, it is unclear whether memory B cells must be measured independently of humoral immunity.

**Figure 2:**
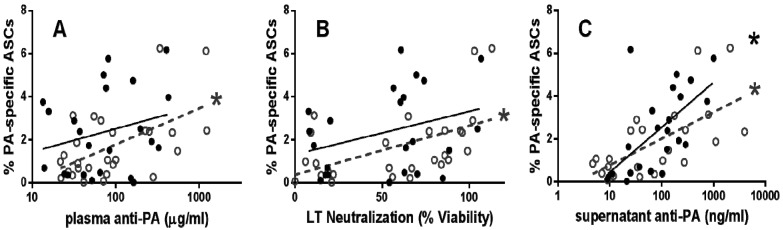
Humoral measures correlate with memory B cell immunity 3–6 months after last vaccination. Plasma anti-PA IgG (**A**) and LT neutralization (**B**) was significantly correlated with PA-specific ASCs in early post-vaccination individuals. In addition, PA-specific ASCs and anti-PA IgG in culture supernatants were correlated in both early and late post-vaccination individuals (**C**). Each symbol represents a single early (open circles) or late (closed circles) post-vaccination individual. All correlations are analyzed by one-tailed Spearman’s correlation (* *p* < 0.05); linear regression lines were added for visualization for both early (dashed line) and late (full line) post vaccination individuals.

In order to assess the potential use of humoral measures as a surrogate for memory B cell immunity, anti-PA IgG and LTNA were assessed for associations with PA-specific ASCs (Figure 2A,B). PA-specific ASC percentages correlated with plasma anti-PA IgG (*r* = 0.42, *p* = 0.03) and LTNA (*r* = 0.52, *p* = 0.003) early post vaccination. In contrast, PA-specific ASCs were not correlated with anti-PA (*r* = 0.21, *p* = 0.17) or LTNA (*r* = 0.24, *p* = 0.14) late post vaccination. Supernatant anti-PA IgG correlated with PA-specific ASCs both early (*r* = 0.60, *p* = 0.001) and late (*r* = 0.71, *p* < 0.0001; Figure 2C) post-vaccination, indicating that the assays for ASC and antibody measurement are consistent. In other studies in which humoral and memory B cell immunity were measured for correlation [5,6,7], samples were not divided by time-post vaccination. Our data suggests that time since last vaccination must be taken into account when determining vaccination response.

## 3. Experimental Section

### 3.1. Collection of Human Blood Samples

Healthy European-American males between the ages of 19 and 45, vaccinated with 3 or more AVA doses or vaccine–naïve (controls *n* = 100), provided written informed consent and a single blood sample. Institutional Review Board approval was obtained from OMRF, OUHSC, WRNMMC, and Womack Army Medical Center. Plasma and PBMCs were isolated from samples and stored at ≤−20 °C or in liquid nitrogen until use.

### 3.2. Anti-PA IgG Concentration and LTNA

ELISAs with recombinant PA and *in vitro* toxin neutralization assays were performed as described [12,13,19,20]. Anti-PA IgG concentration was determined relative to a standard curve of a known reference serum (AVR801, CDC, [21]) in freshly thawed plasma samples and day six ELISPOT culture supernatants. LTNA was determined as the percent viability of macrophages after incubation with LT and a 1:100 dilution of plasma.

### 3.3. Memory B Cell ELISPOT

Memory B cells were quantified by ELISPOT as described [3]. PBMCs were pan-stimulated for six days with pokeweed mitogen extract, CpG (ODN-2006, InvivoGen, San Diego, CA, USA), and fixed *Staphylococcus aureus* (Cowan strain, Sigma, St. Louis, MO, USA). Cultured PBMCs were washed then plated at 1:2 serial dilutions (3.75 × 10^5^ − 1.6 × 10^4^ cells/well) in triplicate onto ELISPOT plates coated with 10 µg/mL PA (List Biologicals, Campbell, CA, USA) or goat anti-human IgG (A80-104A, Bethyl Laboratories, Montgomery, TX, USA). After incubation, plates were incubated HRP-conjugated goat anti-human IgG (Jackson Immunoresearch, 109-036-098; West Grove, PA, USA) and developed with 3 amino-9 ethyl-carbazole (Sigma, St. Louis, MO, USA). Spots in triplicate wells were counted using an Immunospot reader and software (Cellular Technology Limited, Shaker Heights, OH, USA) and averaged. The frequency of PA-specific ASCs was defined as: [(anti-PA IgG spots per 100,000 input cells)/(total IgG spots per 100,000 input cells)] × 100.

## 4. Conclusions

PA-specific antibodies are the strongest correlate of protection against anthrax infection and intoxication in most animal models; unfortunately, anti-PA levels decline rapidly after vaccination. We find that individuals sampled early and late post vaccination with similar levels of plasma anti-PA IgG have similar quantity and quality of PA-specific memory B cells. However, the quantity of circulating PA-specific memory B cells is highly variable, and humoral measures may only serve as a surrogate measure in the first year after vaccination. Indeed, the lack of correlation between memory B cells and antibody late post-vaccination suggests that antibody is maintained independent of memory B cells, likely by long-lived plasma cells. Further, longitudinal studies are necessary to describe the kinetics of the memory B and long-lived plasma cell response in AVA-vaccinated individuals. Regardless of specific kinetics, we demonstrate high levels of PA-specific ASCs exist in the absence of high levels of anti-PA in our late-vaccination group, suggesting that, upon exposure, rapid production of anti-PA IgG could be possible.

## References

[1] Pauli N.T., Henry Dunand C.J., Wilson P.C. (2011). Exploiting human memory B cell heterogeneity for improved vaccine efficacy. Front. Immunol..

[2] Crotty S., Ahmed R. (2004). Immunological memory in humans. Semin. Immunol..

[3] Crotty S., Aubert R.D., Glidewell J., Ahmed R. (2004). Tracking human antigen-specific memory B cells: a sensitive and generalized ELISPOT system. J. Immunol. Methods.

[4] Hammarlund E., Lewis M.W., Hansen S.G., Strelow L.I., Nelson J.A., Sexton G.J., Hanifin J.M., Slifka M.K. (2003). Duration of antiviral immunity after smallpox vaccination. Nat. Med..

[5] Crotty S., Felgner P., Davies H., Glidewell J., Villarreal L., Ahmed R. (2003). Cutting edge: Long-term B cell memory in humans after smallpox vaccination. J. Immunol..

[6] Leyendeckers H., Odendahl M., Lohndorf A., Irsch J., Spangfort M., Miltenyi S., Hunzelmann N., Assenmacher M., Radbruch A., Schmitz J. (1999). Correlation analysis between frequencies of circulating antigen-specific IgG-bearing memory B cells and serum titers of antigen-specific IgG. Eur. J. Immunol..

[7] Ndungu F.M., Olotu A., Mwacharo J., Nyonda M., Apfeld J., Mramba L.K., Fegan G.W., Bejon P., Marsh K. (2012). Memory B cells are a more reliable archive for historical antimalarial responses than plasma antibodies in no-longer exposed children. Proc. Natl. Acad. Sci. USA.

[8] Plotkin S.A. (2008). Vaccines: Correlates of vaccine-induced immunity. Clin. Infect. Dis..

[9] Plotkin S.A. (2010). Correlates of protection induced by vaccination. Clin. Vaccine Immunol..

[10] Reuveny S., White M.D., Adar Y.Y., Kafri Y., Altboum Z., Gozes Y., Kobiler D., Shafferman A., Velan B. (2001). Search for correlates of protective immunity conferred by anthrax vaccine. Infect. Immun..

[11] Quinn C.P., Sabourin C.L., Niemuth N.A., Li H., Semenova V.A., Rudge T.L., Mayfield H.J., Schiffer J., Mittler R.S., Ibegbu C.C. (2012). A three-dose intramuscular injection schedule of anthrax vaccine adsorbed generates sustained humoral and cellular immune responses to protective antigen and provides long-term protection against inhalation anthrax in rhesus macaques. Clin. Vaccine Immunol..

[12] Crowe S.R., Ash L.L., Engler R.J., Ballard J.D., Harley J.B., Farris A.D., James J.A. (2010). Select human anthrax protective antigen epitope-specific antibodies provide protection from lethal toxin challenge. J. Infect. Dis..

[13] Crowe S.R., Garman L., Engler R.J., Farris A.D., Ballard J.D., Harley J.B., James J.A. (2011). Anthrax vaccination induced anti-lethal factor IgG: Fine specificity and neutralizing capacity. Vaccine.

[14] Tross D., Klinman D.M. (2008). Effect of CpG oligonucleotides on vaccine-induced B cell memory. J. Immunol..

[15] Quinn C.P., Dull P.M., Semenova V., Li H., Crotty S., Taylor T.H., Steward-Clark E., Stamey K.L., Schmidt D.S., Stinson K.W. (2004). Immune responses to *Bacillus anthracis* protective antigen in patients with bioterrorism-related cutaneous or inhalation anthrax. J. Infect. Dis..

[16] Goossens P.L. (2009). Animal models of human anthrax: The Quest for the Holy Grail. Mol. Aspects Med..

[17] World Health Organization (WHO) (2004). Anthrax in Humans and Animals.

[18] Allen J.S., Skowera A., Rubin G.J., Wessely S., Peakman M. (2006). Long-lasting T cell responses to biological warfare vaccines in human vaccinees. Clin. Infect. Dis..

[19] Nguyen M.L., Terzyan S., Ballard J.D., James J.A., Farris A.D. (2009). The major neutralizing antibody responses to recombinant anthrax lethal factor and edema factors are directed to non-cross-reactive epitopes. Infect. Immun..

[20] Mohamed N., Li J., Ferreira C.S., Little S.F., Friedlander A.M., Spitalny G.L., Casey L.S. (2004). Enhancement of anthrax lethal toxin cytotoxicity: A subset of monoclonal antibodies against protective antigen increases lethal toxin-mediated killing of murine macrophages. Infect. Immun..

[21] Quinn C.P., Semenova V.A., Elie C.M., Romero-Steiner S., Greene C., Li H., Stamey K., Steward-Clark E., Schmidt D.S., Mothershed E. (2002). Specific, sensitive, and quantitative enzyme-linked immunosorbent assay for human immunoglobulin G antibodies to anthrax toxin protective antigen. Emerg. Infect. Dis..

